# Supplementary material for: Newborn Screening Knowledge, Attitudes and Practices among Obstetrics-Gynecology Residents, Pediatric Residents, and Newborn Screening Nurses in a Tertiary Government Hospital in the Philippines during the COVID-19 Pandemic
Source: Int J Neonatal Screen. 2023 Apr 1;9(2):19. doi: 10.3390/ijns9020019 (PMC10123634; doi:10.3390/ijns9020019)
Supplement: Supplementary file 1 [file IJNS-09-00019-s001.zip › IJNS-2229152-supplementary.pdf]

S1

Knowledge, Attitudes and Practices of Obstetrician-Gynecology Residents, Pediatric Residents, and Newborn Screening Nurses on the Expanded Newborn Screening Program during the COVID-19 Pandemic in a Tertiary Government Hospital

Participant Code: ________

A. Demographics

1. Age: _____

2. Gender:
   a. Male
   b. Female
   c. Prefer not to say

3. Profession (select one)
   a. Pediatric Resident
   b. Obstetrician and Gynecology Resident
   c. Newborn Screening Nurse

4. If you are a Resident, please select your current year level
   a. 1st Year
   b. 2nd Year
   c. 3rd Year
   d. 4th Year

5. If you are a Newborn Screening Nurse, kindly answer the following:
   a. How many years have you been in practice as a nurse? _____
   b. How many years have you been in practice as a Newborn Screening nurse? _____

B. Knowledge Assessment

Please choose the best answer.

1. Newborn Screening (NBS) is a public health program.
   a. True
   b. False

2. NBS requires consent from the parent.
   a. True
   b. False

3. The disease screened in the NBS vary for each region in the Philippines
    a. True
    b. False

4. When should NBS ideally be performed for a term healthy infant?
    a. Right away after birth
    b. During the first 24 hours of life
    c. Immediately 24 hours from birth
    d. Anytime within the neonatal period

5. NBS is usually done by
    a. Heel prick
    b. Finger prick
    c. Arterial blood
    d. Any of the above

6. The current Expanded NBS screens for how many disorders:
    a. 6
    b. 9
    c. 12
    d. More than 20

7. If the baby looks normal, he or she does not have any congenital disorder.

a. True
b. False

8. Any trained health personnel can collect blood samples of the baby.

a. True
b. False

9. With regards the Expanded Newborn Screening results, which statement is correct?

a. It will be available after 24 hours
b. Mother is contacted when there is a positive screen
c. After 1 week
d. At first well baby check up

10. Further confirmatory testing is not needed if the result is a positive screen.

a. True
b. False

11. Babies with positive results must be referred for confirmatory testing and further management.
    a. True
    b. False

12. The Expanded NBS panel includes:
    a. Assessing red reflex, pulse oximetry, transcutaneous bilirubin
    b. Autism, hearing screening, critical congenital heart disease
    c. G6PD deficiency, Congenital adrenal hyperplasia, cystic fibrosis, thalassemia are some of the conditions
    d. None of the above

13. The following information are important for NBS:
    a. Date and time of birth and date and time of specimen collection
    b. Gestational age and birth weight
    c. Information on feeding, i.e. baby on total parenteral nutrition
    d. all of the above

NBS and COVID

14. Expanded Newborn Screening services are deferred during the COVID-19 pandemic.
    a. True
    b. False

15. Personal protective equipment are worn when extracting blood samples from newborns whose mothers are suspected or confirmed COVID-19 cases.
    a. True
    b. False

16. Samples are preferably placed under an aerosol box for drying.
    a. True
    b. False

17. Drying a blood sample should be done in a safe, clean and dry place, and away from personnel traffic flow.
    a. True
    b. False

18. Drying of cards whose mothers are suspected or confirmed COVID-19 cases do not need to be separated from the other cards
    a. True
    b. False

19. Packing of cards of newborns of suspected/confirmed COVID-19 mothers do not need to be separated from the rest.
    a. True
    b. False

20. Cards should be labeled appropriately as COVID-19 suspect, COVID-19 probable, or COVID-19 positive mother.
    a. True
    b. False

21. In the laboratories, proper protective equipment should be worn when sorting, punching, and running of samples.
    a. True
    b. False

Attitudes Assessment

Please choose one option for each statement.

1. Newborn screening is a useful tool for identifying certain conditions that if undiagnosed can cause some serious problems, such as brain damage, organ damage, and even death
    a. Strongly disagree
    b. Somewhat disagree
    c. Neutral
    d. Somewhat agree
    e. Strongly agree

2. A patient can still have the disease/disorder even if the NBS is negative
    a. Strongly disagree
    b. Somewhat disagree
    c. Neutral
    d. Somewhat agree
    e. Strongly agree

3. I am comfortable counseling the parents if screen tests reveal a positive screening result
    a. Strongly disagree
    b. Somewhat disagree
    c. Neutral
    d. Somewhat agree
    e. Strongly agree

4. Pediatricians play a central role in ensuring that their patients benefit from early detection of a positive screen or early detection of a disorder
    a. Strongly disagree
    b. Somewhat disagree
    c. Neutral
    d. Somewhat agree
    e. Strongly agree

5. Obstetricians-Gynecologists play a central role in ensuring that their patients benefit from early detection of a positive screen
    a. Strongly disagree
    b. Somewhat disagree
    c. Neutral
    d. Somewhat agree
    e. Strongly agree

6. Newborn Screening Nurses play a central role in ensuring that their patients benefit from early detection of a positive screen
    a. Strongly disagree
    b. Somewhat disagree
    c. Neutral
    d. Somewhat agree
    e. Strongly agree

7. I need more training in the area of the newborn screening
    a. Strongly disagree
    b. Somewhat disagree
    c. Neutral
    d. Somewhat agree
    e. Strongly agree

7a. If you answered strongly/somewhat agree to the previous question, what aspect of NBS do you need training on? ______________

8. Newborn Screening is worth the discomfort the baby feels when pricked
    a. Strongly disagree
    b. Somewhat disagree
    c. Neutral
    d. Somewhat agree
    e. Strongly agree
9. I will share information regarding newborn screening to other parents and/or peers
    a. Strongly disagree
    b. Somewhat disagree
    c. Neutral
    d. Somewhat agree
    e. Strongly agree
10. Newborn Screening is still necessary even if the baby appears healthy at birth
    a. Strongly disagree
    b. Somewhat disagree
    c. Neutral
    d. Somewhat agree

e. Strongly agree

NBS and COVID-19

11. Newborn Screening services can be postponed during the time of the COVID-19 pandemic
    a. Strongly disagree
    b. Somewhat disagree
    c. Neutral
    d. Somewhat agree
    e. Strongly agree

12. Safety of the newborn is compromised when newborn screening is done during the time of the COVID-19 pandemic
    a. Strongly disagree
    b. Somewhat disagree
    c. Neutral
    d. Somewhat agree
    e. Strongly agree

13. Newborn screening services can be continued provided that proper personal protective equipment is used by the healthcare worker
    a. Strongly disagree
    b. Somewhat disagree
    c. Neutral
    d. Somewhat agree
    e. Strongly agree

14. I would like to learn more information about newborn screening during the time of the COVID-19 pandemic
    a. Strongly disagree
    b. Somewhat disagree
    c. Neutral
    d. Somewhat agree
    e. Strongly agree
15. I will encourage other parents to have their child undergo newborn screening even during the time of the COVID-19 pandemic
    a. Strongly disagree
    b. Somewhat disagree
    c. Neutral
    d. Somewhat agree
    e. Strongly agree

Practice Assessment

Please choose one option for each question in each clinical setting

1.

For OB-GYNS: How often do you discuss the rationale for doing NBS with the mother/parents/guardians during prenatal consults (whether face-to-face or telemedicine consultations)?

For Pedia: How often do you discuss the rationale for doing NBS with the mother/parents/guardians during patient interactions (whether face-to-face or telemedicine consultations)?

For nurses: How often do you discuss the rationale for doing NBS with the mother/parents/guardians during patient interactions?

a. 76%-100% of time
b. 51-75% of the time
c. 26%-50% of the time
d. 1-25% of the time
e. 0% of the time: please indicate if anybody else does: ___________

2. How often do you discuss the rationale for doing NBS with the mother/parents/guardians during the admission of the mother prior to giving birth?
   a. 76%-100% of time
   b. 51-75% of the time
   c. 26%-50% of the time
   d. 1-25% of the time
   e. 0% of the time: please indicate if anybody else does: ___________
3. How often do you discuss the rationale for doing NBS with the mother/parents/guardians after the mother gives birth?
   a. 76%-100% of time
   b. 51-75% of the time
   c. 26%-50% of the time
   d. 1-25% of the time
   e. 0% of the time: please indicate if anybody else does: ___________
4. How often do you actively seek the results of NBS?
   a. 76%-100% of time
   b. 51-75% of the time
   c. 26%-50% of the time
   d. 1-25% of the time
   e. 0% of the time: please indicate if anybody else does: ___________
5. How often do you inform parents/guardians if results are reported normal?
   a. 76%-100% of time

    b. 51-75% of the time
    c. 26%-50% of the time
    d. 1-25% of the time
    e. 0% of the time: please indicate if anybody else does: ___________
6. Do you know the agency to contact for NBS results>
    a. Yes. If yes please indicate what agency: ______
    b. No.
7. If screening is positive, are you aware of the appropriate protocol for follow-up?
    a. Yes
    b. No
    c. Not sure
    d. Never been in this situation

Open ended questions for the participants:

1. Which among the procedures in NBS flow at the hospital level (i.e. motivating parents, taking sample immediately after 24 hours, taking quality samples, sending, releasing of results on time or immediately if positive, referring to confirmatory if positive) are not properly or strictly followed during CoVID-19 pandemic?

2. What are your challenges in implementing Expanded Newborn Screening in PGH during the COVID-19 pandemic?

3. What are your suggestions to improve Expanded Newborn Screening services delivery during the COVID-19 pandemic?

S2. Participants' level of Knowledge on NBS and Knowledge on NBS and COVID

**Category of Health Worker**	**Total Score** Mean (21/21)	**Knowledge on NBS** (Score for Questions 1-13) Mean	**Knowledge on NBS and COVID-19** (Score for Questions 14-21) Mean
Pediatric Resident	17.34	11.09	6.25
Obstetrics and Gynecology Resident	16.61	9.88	6.73
Newborn Screening Nurse	17.47	11.15	6.32

S3. Participants scores on Knowledge with MPL applied

	**Pediatric Residents N=55**	**OB-GYN Residents N=49**	**NBS Nurses N=85**
Favorable ≥ MPL	45 (81.82%)	36 (73.47%)	73 (85.88%)
Not Favorable < MPL	10 (18.18%)	13 (26.53%)	12 (14.12%)

S4. Breakdown of Participants with Correct Answers to Knowledge Questions on ENBS

Questions	Category of Health Worker			
	Pediatric Resident N= 55 No (%)	OB-Gyn Resident N= 49 No (%)	NBS Nurse N= 85 No (%)	Overall N= 189 No (%)
NBS in General<br>1. NBS is a public health program. (Ans. True)	54 (98.18)	49 (100.00)	83 (97.65)	186(98.41)
2. NBS requires consent from the parent. (Ans. False)	26 (47.27)	14 (28.57)	31(36.47)	71 (37.57)
3. The diseases screened in the NBS vary for each region in the Philippines. (Ans. False)	46 (83.64)	45 (91.84)	71 (83.53)	162 (85.71)
4. When should NBS ideally be performed for a term healthy infant? (Ans. Immediately 24 hours after birth)	43 (78.18)	22 (44.90)	64 (75.29)	129 (68.25)
5. NBS is usually done by (Ans. Heelprick)	55 (100.00)	49 (100.00)	85 (100.00)	189 (100.00)
6. The current Expanded NBS screens for how many disorders: (Ans. More than 20)	72 (76.36)	16 (32.65)	72 (84.71)	130 (68.78)
7. If the baby looks normal, he or she does not have any congenital disorder. (Ans. False)	54 (98.18)	49 (100.00)	84 (98.82)	187 (98.94)
8. Any trained health personnel can collect blood samples of the baby (Ans. True)	38 (69.09)	38 (77.55)	59 (69.41)	135 (71.43)
9. With regards the ENBS results, which statement is correct? (Ans. Mother is contacted when there is a positive screen)	49 (89.09)	29 (59.18)	77 (90.59)	155 (82.01)
10. Further confirmatory testing is not needed if the result is a positive screen. (Ans. False)	48 (87.27)	42 (85.71)	77 (90.59)	167 (88.36)
11. Babies with positive results must be referred for confirmatory testing and further management. (Ans. True)	55 (100.00)	49 (100.00)	85 (100.00)	189 (100.00)
12. The ENBS panel includes:<br>(Ans. G6PD deficiency, Congenital Adrenal Hyperplasia, Cystic Fibrosis, Thalassemia are some of the conditions)	52 (94.55)	41 (83.67)	76 (89.41)	169 (89.42)
13. The following information are important for NBS: (Ans. Date/time of birth, time specimen collection, gestational age and birth weight, feeding history)	48 (87.27)	41 (83.67)	84 (98.82)	173 (91.53)
NBS and COVID<br>14. ENBS services are deferred during the COVID-19 pandemic.(Ans. False)	54 (98.18)	48 (97.96)	81 (95.29)	182 (96.83)
15. Personal protective equipment are worn when extracting blood samples from newborns whose mothers are suspected or confirmed COVID-19 cases. (Ans. True)	55 (100.00)	45 (91.84)	82 (96.47)	182 (96.30)
16. Samples are preferably placed under an aerosol box for drying. (Ans. True)	42 (76.36)	39 (79.59)	46 (54.12)	127 (67.20)
17. Drying a blood sample should be done in a safe, clean and dry place, and away from personnel traffic flow. (Ans. True)	55 (100.00)	47 (95.92)	85 (100.00)	187 (98.94)
18. Drying of cards whose mothers are suspected or confirmed COVID-19 cases do not need to be separated from the other cards (Ans. False)	22 (40.00)	28 (57.14)	46 (54.12)	96 (50.79)
19. Packing of cards of newborns of suspected/confirmed COVID-19 mothers do not need to be separated from the rest. (Ans. False)	25 (45.45)	34 (69.39)	49 (57.65)	108 (57.14)
20. Cards should be labeled appropriately as COVID-19 suspect, COVID-19 probable, or COVID-19 positive mother. (Ans True)	40 (72.73)	41 (83.67)	68 (80.00)	149 (78.84)

21. In the laboratories, proper protective equipment should be worn when sorting, punching, and running of samples. (Ans. True)	51 (92.73)	48 (97.96)	80 (94.12)	179 (94.71)

S5. Participants scores on Attitude with MPL applied

	**Pediatric Residents** N=55	**OB-GYN Residents** N=49	**NBS Nurses** N=85
Favorable (Score $\geq$ MPL)	52 (94.55%)	45 (91.84%)	76 (89.41%)
Not Favorable (Score < MPL)	3 (5.45%)	4 (8.16%)	9 (10.59%)

S6. Breakdown of Participants' response to attitude items on ENBS

**Questions**	**Category of Health Worker**			**Overall** N = 189 No (%)
	**Pediatric Resident** N= 55 No (%)	**OB-Gyn Resident** N= 49 No (%)	**NBS Nurse** N= 85 No (%)	
1. Newborn screening is a useful tool for identifying certain conditions if undiagnosed can cause some serious problems, such as brain damage, organ damage, and even death				
a. Strongly disagree				
b. Somewhat disagree	3 (5.4)	5 (10.2)	17 (20.0)	25 (13.2)
c. Neutral	0	0	0	0
d. Somewhat agree	0	0	1 (1.2)	1 (0.5)
e. Strongly agree	0	2 (4.1)	0	2 (1.1)
	52 (94.6)	42 (85.7)	67 (78.8)	161 (85.2)
2. A patient can still have the disease/disorder even if the NBS is negative				
a. Strongly disagree	4 (7.3)	3 (6.1)	20 (23.5)	27 (14.3)
b. Somewhat disagree	6 (10.9)	7 (14.3)	11 (12.9)	24 (12.7)
c. Neutral	3 (5.4)	8 (16.3)	12 (14.1)	23 (12.2)
d. Somewhat agree	13 (23.6)	17 (34.7)	16 (18.8)	46 (24.3)
e. Strongly agree	29 (52.7)	14 (28.6)	26 (30.6)	69 (36.5)
3. I am comfortable counseling the parents if screen tests reveal a positive screening result				
a. Strongly disagree	2 (3.6)	2 (4.1)	5 (5.9)	9 (4.8)
b. Somewhat disagree	3 (5.4)	9 (18.4)	6 (7.1)	18 (9.5)
c. Neutral	8 (14.6)	17 (34.7)	13 (15.3)	38 (20.1)
d. Somewhat agree	28 (50.9)	16 (32.7)	26 (30.6)	70 (37.0)
e. Strongly agree	14 (25.4)	5 (10.2)	35 (41.2)	54 (28.6)
4. Pediatricians play a central role in ensuring that their patients benefit from early detection of a positive screen or early detection of a disorder				
a. Strongly disagree	3 (5.4)	1 (2.0)	5 (5.9)	9 (4.8)
b. Somewhat disagree	0	0	0	0
c. Neutral	0	1 (2.0)	0	1 (0.5)
d. Somewhat agree	2 (3.6)	7 (14.3)	4 (4.7)	13 (6.88)
e. Strongly agree	50 (90.9)	40 (81.6)	76 (89.4)	166 (87.83)
5. Obstetricians-Gynecologists play a central role in ensuring that their patients benefit from early detection of a positive screen				
a. Strongly disagree	3 (5.4)	0	4 (4.7)	7 (3.7)
b. Somewhat disagree	0	2 (4.1)	1 (1.2)	3 (1.6)

c. Neutral	0	7 (14.3)	5 (5.9)	12 (6.3)
d. Somewhat agree	10 (18.2)	18 (36.7)	9 (10.6)	37 (19.58)
e. Strongly agree	42 (76.4)	22 (44.9)	66 (77.6)	130 (68.78)
6. Newborn Screening Nurses play a central role in ensuring that their patients benefit from early detection of a positive screen				
a. Strongly disagree	3 (5.4)	1 (2.0)	6 (7.1)	10 (5.3)
b. Somewhat disagree	0	0	0	0
c. Neutral	0	3 (6.1)	1 (1.2)	4 (2.1)
d. Somewhat agree	3 (5.4)	14 (28.6)	6 (7.1)	23 (12.17)
e. Strongly agree	49 (89.1)	31 (63.3)	72 (84.7)	152 (80.42)
7. I need more training in the area of the newborn screening				
a. Strongly disagree				
b. Somewhat disagree	1 (1.8)	0	4 (4.7)	5 (2.6)
c. Neutral	2 (3.6)	0	4 (4.7)	6 (3.2)
d. Somewhat agree	18 (32.7)	16 (32.6)	35 (41.2)	69 (33.3)
e. Strongly agree	18 (32.7)	17 (34.7)	20 (23.5)	55 (29.1)
	16 (29.1)	16 (32.6)	22 (25.9)	54 (28.6)
8. Newborn Screening is worth the discomfort the baby feels when pricked				
a. Strongly disagree	1 (1.8)	0	3 (3.5)	4 (2.1)
b. Somewhat disagree	1 (1.8)	0	1 (1.2)	2 (1.1)
c. Neutral	1 (1.8)	2 (4.08)	2 (2.4)	5 (2.6)
d. Somewhat agree	6 (10.9)	11 (22.4)	17 (20.0)	34 (18.0)
e. Strongly agree	46 (83.6)	36 (73.5)	62 (72.9)	144 (76.2)
9. I will share information regarding newborn screening to other parents and/or peers				
a. Strongly disagree	3 (5.4)	1 (2.0)	10 (11.8)	14 (7.4)
b. Somewhat disagree	3 (5.4)	1 (2.0)	0	4 (2.1)
c. Neutral	0	0	0	0
d. Somewhat agree	6 (10.9)	16 (32.6)	5 (9.4)	30 (15.9)
e. Strongly agree	43 (78.2)	31 (63.3)	67 (78.8)	141 (74.6)
10. Newborn Screening is still necessary even if the baby appears healthy at birth				
a. Strongly disagree	1 (1.8)	0	2 (2.4)	3 (1.6)
b. Somewhat disagree	0	0	0	0
c. Neutral	0	0	0	0
d. Somewhat agree	0	1 (2.04)	0	1 (0.5)
e. Strongly agree	54 (98.2)	48 (98.0)	83 (97.6)	185 (97.9)
11. Newborn Screening services can be postponed during the time of the COVID-19 pandemic				
a. Strongly disagree	51 (92.7)	6 (12.2)	63 (74)	120 (63.5)
b. Somewhat disagree	4 (7.3)	17 (34.7)	11 (12.94)	32 (16.9)
c. Neutral	0	6 (12.2)	1 (1.2)	7 (3.7)
d. Somewhat agree	0	3 (6.1)	1 (1.2)	4 (2.1)
e. Strongly agree	0	17 (34.7)	9 (10.6)	26 (13.8)
12. Safety of the newborn is compromised when newborn screening is done during the time of the COVID-19 pandemic				
a. Strongly disagree				
b. Somewhat disagree	44 (80.0)	21 (42.8)	50 (58.8)	115 (60.8)
c. Neutral	8 (14.6)	20 (40.8)	11 (12.9)	39 (20.6)
d. Somewhat agree	1 (1.8)	4 (8.2)	10 (11.8)	15 (7.9)
e. Strongly agree	1 (1.8)	1 (2.0)	7 (8.2)	9 (4.8)
	1 (1.8)	3 (6.1)	7 (8.2)	11 (5.8)
13. Newborn screening services can be continued provided that proper personal protective equipment is used by the healthcare worker				
a. Strongly disagree	2 (3.6)	0	3 (3.5)	5 (2.7)

b. Somewhat disagree	0	0	1 (1.2)	1 (0.5)
c. Neutral	0	2 (4.1)	2 (2.4)	4 (2.1)
d. Somewhat agree	5 (9.1)	4 (8.2)	7 (8.2)	16 (8.47)
e. Strongly agree	48 (87.3)	43 (87.8)	72 (84.7)	163 (86.2)
14. I would like to learn more information about newborn screening during the time of the COVID-19 pandemic				
a. Strongly disagree	2 (3.6)	0	0	2 (1.1)
b. Somewhat disagree	0	0	0	0
c. Neutral	3 (5.4)	5 (10.2)	7 (8.2)	15 (7.9)
d. Somewhat agree	14 (25.4)	16 (32.6)	16 (18.8)	46 (24.3)
e. Strongly agree	36 (65.4)	28 (57.1)	62 (72.9)	126 (66.7)
15. I will encourage other parents to have their child undergo newborn screening even during the time of the COVID-19 pandemic				
a. Strongly disagree	2 (3.6)	0	3 (3.5)	5 (2.6)
b. Somewhat disagree	0	0	0	0
c. Neutral	0	0	0	0
d. Somewhat agree	0	6 (12.2)	7 (8.2)	13 (6.9)
e. Strongly agree	53 (96.4)	43 (87.8)	75 (88.2)	171 (90.5)

S7 Breakdown of Participants' response to practice items on ENBS

	No (%) of Pediatric Residents N = 55	No (%) of OB-GYN Residents N = 49	No (%) of NBS Nurse N = 85	Overall No (%) N=189
1. How often do you discuss the rationale for doing NBS with the mother/parents/guardians during prenatal consults (whether face-to-face or telemedicine consultations)?				
a. 76%-100% of time	16 (29.09)	3 (6.12)	44 (51.76%)	63 (33.33)
b. 51-75% of the time	22 (40.00)	4 (8.16)	14 (16.47)	40 (21.16)
c. 26%-50% of the time	12 (21.82)	6 (12.24)	17 (20.00)	35 (18.52)
d. 1-25% of the time	5 (9.09)	19 (38.78)	9 (10.59)	33 (17.46)
e. 0% of the time	0 (0.00)	17 (34.69)	1 (1.18)	18 (9.52)
2. How often do you discuss the rationale for doing NBS with the mother/parents/guardians during the admission of the mother prior to giving birth?				
a. 76%-100% of time	14 (25.45)	1 (2.04)	20 (23.53)	35 (18.52)
b. 51-75% of the time	20 (36.36)	5 (10.20)	16 (18.82)	41 (21.69)
c. 26%-50% of the time	11 (20.00)	7 (14.29)	18 (21.18)	36 (19.05)
d. 1-25% of the time	7 (12.73)	19 (38.78)	14 (16.47)	40 (21.16)
e. 0% of the time	3 (5.45)	17 (34.69)	17 (20.00)	37 (19.58)
3. How often do you discuss the rationale for doing NBS with the mother/parents/guardians after the mother gives birth?				
a. 76%-100% of time	23 (41.82)	3 (6.12)	40 (47.06)	66 (34.92)
b. 51-75% of the time	19 (34.55)	9 (18.37)	13 (15.29)	41 (21.69)
c. 26%-50% of the time	9 (16.36)	5 (10.20)	14 (16.47)	28 (14.81)
d. 1-25% of the time	4 (7.27)	19 (38.78)	11 (12.94)	34 (17.99)
e. 0% of the time	0 (0.00)	13 (26.53)	7 (8.24)	20 (10.58)
4. How often do you actively seek the results of NBS?				
a. 76%-100% of time	22 (40.00)	1 (2.04)	36 (42.35)	59 (31.22)
b. 51-75% of the time	15 (27.27)	2 (4.08)	18 (21.18)	35 (18.52)
c. 26%-50% of the time	11 (20.00)	2 (4.08)	15 (17.65)	28 (14.81)
d. 1-25% of the time	5 (9.09)	20 (40.82)	12 (14.12)	37 (19.58)
e. 0% of the time	2 (3.64)	24 (48.98)	4 (4.71)	30 (15.87)

5. How often do you inform parents/guardians if results are reported normal?				
a. 76%-100% of time	11 (20.00)	1 (2.04)	36 (42.35)	59 (31.22)
b. 51-75% of the time	16 (29.09)	1 (2.04)	18 (21.18)	35 (18.52)
c. 26%-50% of the time	13 (23.64)	2 (4.08)	15 (17.65)	28 (14.81)
d. 1-25% of the time	10 (18.18)	12 (24.49)	12 (14.12)	37 (19.58)
e. 0% of the time	5 (9.09)	33 (67.35)	4 (4.71)	30 (15.87)
6. Do you know the agency to contact for NBS results?				
a. Yes	44 (80.00)	9 (18.37)	83 (97.65)	136 (71.96)
b. No	11 (20.00)	40 (81.63)	2 (2.35)	53 (28.04)
7. If screening is positive, are you aware of the appropriate protocol for follow-up?				
a. Yes	33 (60.00)	2 (4.08)	74 (87.06)	109 (57.67)
b. No	4 (7.27)	15 (30.61)	0 (0.00)	19 (10.05)
c. Not sure	14 (25.45)	15 (30.61)	6 (7.06)	35 (18.52)
d. Never been in this situation	4 (7.27)	17 (34.69)	5 (5.88)	26 (13.76)

S8 Participants scores on Practice with MPL applied

	**Pediatric Residents** N=55	**OB-GYN Residents** N=49	**NBS Nurses** N=85
Favorable (Score ≥ MPL)	16 (29.09%)	2 (4.08%)	40 (47.06%)
Not Favorable (Score < MPL)	39 (70.91%)	47 (96.92%)	45 (52.94%)

S9 Results of the Multiple Linear Regression Analysis, Outcome: Knowledge Score

**High Knowledge Score**	**Adjusted Odds Ratio**	**Std Error**	**P-value**	**95% Confidence Interval**	
				Lower	Upper
Age, years	1.03	1.30	0.477	0.948	1.119
Sex (compared to males)					
Female	0.27	0.213	0.097	0.057	1.270
Other/NI	0.06	0.104	0.091	0.003	1.556
Profession (compared to Pedia Residents)					
OB-Gyn Residents	0.70	0.345	0.466	0.264	1.841
Nurses	0.92	0.590	0.893	0.259	3.243
Years in Profession (years of service in one's profession)	1.07	0.081	0.338	0.927	1.246

S10 Results of the Multiple Logistic Regression Analysis, Outcome: High Knowledge Score

**High Knowledge Score**	**Adjusted Odds Ratio**	**Std Error**	**P-value**	**95% Confidence Interval**	
				Lower	Upper
Age, years	1.03	1.30	0.477	0.948	1.119
Sex (compared to males)					
Female	0.27	0.213	0.097	0.057	1.270
Other/NI	0.06	0.104	0.091	0.003	1.556
Profession (compared to Pedia Residents)					

OB-Gyn Residents	0.70	0.345	0.466	0.264	1.841
Nurses	0.92	0.590	0.893	0.259	3.243
Years in Profession (years of service in one's profession)	1.07	0.081	0.338	0.927	1.246

S11 Results of the multiple linear regression, Outcome: Practice MPL

**Practice MPL**	**Adjusted Coefficient**	**Std Error**	**P-value**	**95% Confidence Interval**	
				Lower	Upper
Age, years	0.972	0.030	0.363	0.914	1.033
Sex (compared to males)					
Female	0.730	0.423	0.588	0.234	2.276
Other/NI	1.015	1.604	0.992	0.045	22.477
Profession (compared to Pedia Residents)					
OB-Gyn Residents	0.111	0.088	0.006	0.023	0.525
Nurses	2.694	1.463	0.068	0.028	7.814
Years in Profession (years of service in one's profession)	1.045	0.048	0.339	0.954	1.145
Knowledge MPL	1.375	0.705	0.535	0.503	3.757
Attitude MPL	0.580	0.389	0.417	0.155	16.92
